# Supplementary material for: Cadmium-induced DAG-PKC/IP3-Ca2+ double signaling pathway and cell injury in gill of freshwater crabs Sinopotamon henanense
Source: PLoS One. 2026 Apr 22;21(4):e0336415. doi: 10.1371/journal.pone.0336415 (PMC13102191; doi:10.1371/journal.pone.0336415)
Supplement: Attachment — Submitted filename: Response to editor.doc [file pone.0336415.s003.doc]

Dear Editor,

Thank you for sending us the useful comments. We have carefully revised our manuscript according the comments provided by the editor. Details on the revisions are listed below.

1. Please include your tables as part of your main manuscript and remove the individual files. Please note that supplementary tables (should remain/ be uploaded) as separate "Supporting Information" files.

**Answer**: we have included table 1 in the manuscript and remove the individual files.

2. Please include a separate legend for each figure in your manuscript.

**Answer**: we have included figure legends in the manuscript.

3. We note that your author list was updated during the revision process. In order to add or remove authors or update the order of the author byline after initial submission, we ask that authors complete an Authorship Change Request form.
**Answer**: We sincerely apologize for any inconvenience caused by the update to the author list. The changes to the authorship were made to ensure that the byline accurately reflects the contributions of all individuals involved in this work. All authors have discussed and unanimously agreed to these changes, and there are no conflicts among us regarding this update. We have completed the required Authorship Change Request form and attached it to this email for your review.

4. We note that your Data Availability Statement is currently as follows: All relevant data are within the manuscript and in Supporting Information files.

Please confirm at this time whether or not your submission contains all raw data required to replicate the results of your study. Authors must share the “minimal data set” for their submission. PLOS defines the minimal data set to consist of the data required to replicate all study findings reported in the article, as well as related metadata and methods (https://journals.plos.org/plosone/s/data-availability#loc-minimal-data-set-definition).

**Answer**: We have included all the experimental data mentioned in the manuscript in a file named "Experimental data"and attached it to this email for your review.

Please let us know if you require any additional information or further clarification. We greatly appreciate your understanding and assistance in this matter.

Thank you for your time and consideration.

Best regards,

Wang Jinxiang
